# Depressive and anxiety disorders and antidepressant prescriptions among insured children and young adults with congenital adrenal hyperplasia in the United States

**DOI:** 10.3389/fendo.2023.1129584

**Published:** 2023-08-17

**Authors:** Lauren A. Harasymiw, Scott D. Grosse, Kathryn R. Cullen, Rebecca H. Bitsko, Ruth Perou, Kyriakie Sarafoglou

**Affiliations:** ^1^ Department of Pediatrics, University of Minnesota Medical School, Minneapolis, MN, United States; ^2^ National Center on Birth Defects and Developmental Disabilities, Centers for Disease Control and Prevention, Atlanta, GA, United States; ^3^ Department of Psychiatry and Behavioral Science, University of Minnesota Medical School, Minneapolis, MN, United States; ^4^ Department of Pediatrics, Division of Pediatric Endocrinology, University of Minnesota Medical School, Minneapolis, MN, United States; ^5^ Department of Experimental and Clinical Pharmacology, University of Minnesota College of Pharmacy, Minneapolis, MN, United States

**Keywords:** congenital adrenal hyperplasia, depression, anxiety, antidepressants, glucocorticoids

## Abstract

**Background:**

Dysfunction in the hypothalamic-pituitary-adrenal axis has been associated with depressive and anxiety disorders. Little is known about the risk for these disorders among individuals with congenital adrenal hyperplasia (CAH), a form of primary adrenal insufficiency.

**Objective:**

We investigated the prevalence of depressive and anxiety disorders and antidepressant prescriptions in two large healthcare databases of insured children, adolescents, and young adults with CAH in the United States.

**Methods:**

We conducted a retrospective cohort study using administrative data from October 2015 through December 2019 for individuals aged 4–25 years enrolled in employer-sponsored or Medicaid health plans.

**Results:**

Adjusting for age, the prevalence of depressive disorders [adjusted prevalence ratio (aPR) = 1.7, 95% confidence interval (CI): 1.4-2.0, p<0.001], anxiety disorders [aPR = 1.7, 95% CI: 1.4-1.9, p<0.001], and filled antidepressant prescriptions [aPR = 1.7, 95% CI: 1.4-2.0, p<0.001] was higher among privately insured youth with CAH as compared to their non-CAH peers. Prevalence estimates were also higher among publicly insured youth with CAH for depressive disorders [aPR = 2.3, 95% CI: 1.9-2.9, p<0.001], anxiety disorders [aPR = 2.0, 95% CI: 1.6-2.5, p<0.001], and filled antidepressant prescriptions [aPR = 2.5, 95% CI: 1.9-3.1, p<0.001] as compared to their non-CAH peers.

**Conclusions:**

The elevated prevalence of depressive and anxiety disorders and antidepressant prescriptions among youth with CAH suggests that screening for symptoms of depression and anxiety among this population might be warranted.

## Introduction

Congenital adrenal hyperplasia (CAH) is an inherited form of primary adrenal insufficiency characterized by impaired cortisol synthesis and increased adrenal androgen production (1). Classic CAH requires life-long glucocorticoid replacement (2). Individuals with the salt-wasting form of classic CAH may also require mineralocorticoid replacement to counteract aldosterone deficiency (2). Non-classic CAH is a milder disorder with normal cortisol production in most cases; individuals with non-classic CAH typically require glucocorticoid replacement only when symptomatic (3). In the United States, the frequency of CAH detected by newborn screening programs ranges from approximately 1 in 16,000 to 1 in 18,000 (4, 5). Non-classic CAH is more common, with an estimated prevalence of approximately 1 in 200 US adults of European ancestry (6).

During childhood, hydrocortisone, a short-acting glucocorticoid, is used for replacement therapy to minimize the adverse impact on growth from long‐acting glucocorticoids. Due to its short half-life, treatment with hydrocortisone can lead to alternating states of hypercortisolemia and hypocortisolemia with resultant hyperandrogenemia (7, 8). Chronic hypercortisolemia can lead to growth failure, iatrogenic Cushing syndrome, hypertension, increased weight gain, infertility, metabolic syndrome, hypertension, and osteoporosis in adulthood (9–13). Chronic hypocortisolemia and exposure to excess androgen can lead to virilization, peripheral precocious puberty, advanced bone age, growth acceleration and early epiphyseal closure leading to short stature (1, 10, 14).

The potential effects of CAH on mental health, either due to coping with a life-long disease or from the inherent hypothalamic-pituitary-adrenal axis dysfunction and associated therapeutic limitations, are less well understood. Some evidence suggests that individuals with CAH are more likely to experience symptoms of depression or anxiety compared to the general population (15–17). In a recent matched-cohort study from the United Kingdom using a primary-care based administrative database (UK Clinical Practice Research Datalink), Jenkins-Jones and colleagues (17) reported a higher prevalence of depression among 255 CAH patients younger than 18 years old, but no difference in the lifetime prevalence of depression diagnoses or antidepressant use among individuals aged 18–40 years. Two other recent studies utilized a national patient registry in Sweden to evaluate mental health diagnoses in large samples of patients with CAH (18, 19). Engberg and colleagues (18) reported that the odds ratio of a mood or anxiety disorder was 1.7 for females over the age of 18 years with CAH in Sweden compared to females the same age without CAH. However, there was no increase in risk of those diagnoses among females 12–18 years old with CAH, although substance misuse was significantly elevated among both adolescents and adults with CAH. Falhammar and colleagues (19) reported statistically insignificant odds ratios of 1.6 and 1.2 for mood and anxiety disorders for 239 males with CAH (median age 23.2 years) in Sweden compared to age-matched controls from the general population. Because they did not report age-stratified analyses, it is difficult to know how these findings apply to the pediatric population.

In particular, there is a paucity of data describing the prevalence of diagnosed depressive and anxiety disorders among large cohorts of pediatric and young adult patients with CAH. Most prior pediatric studies have reported information on relatively small samples of children and adolescents with CAH and utilized behavioral scales to measure parent-reported symptoms (20–23). For instance, Messina and colleagues conducted an observational study involving Swedish and Italian children aged 7-17 years with CAH (n = 57). They found that parents of CAH patients rated their children as having more social problems, as measured by the Child Behavior Checklist (CBCL), compared to the control group. No differences in internalizing problems or anxiety/depression symptoms were reported (20–23). In contrast, Idris and colleagues reported that parents disclosed a higher rate of both internalizing and externalizing problems among children aged 6-18 years with CAH in Malaysia (n = 49) as compared to a control group made up of non-affected relatives. However, there was no difference in the proportion of children with CAH with clinically significant scores on the anxious/depressed or withdrawn/depression syndrome subscales of the CBCL (20–23). In a study of 81 children ages 4-11 years with CAH in the United Kingdom, Kung and colleagues using found increased scores for conduct problems and hyperactivity/inattention and lower scores for pro-social behaviors via parent-report on the Strengths and Difficulties Questionnaire for girls with CAH as compared to unaffected relatives. No differences in emotional symptoms were noted for boys or girls, either as compared to unaffected relatives or the general population (20–23). Finally, in a study of 114 children and young adults with CAH (ages 3-31 years) in the United States, Berenbaum and colleagues did not find any differences in parent-report of internalizing or behavioral problems as measured by the CBCL among boys or girls CAH as compared to unaffected relatives. However, a more negative affect was noted for adolescent and adults males with CAH as measured by the Self-Image Questionnaire for Young Adolescents (20–23).

The current study aimed to investigate the prevalence of depressive and anxiety disorders and antidepressant prescriptions among insured children, adolescents, and young adults in the United States with and without CAH who were enrolled in health plans contributing records to one of two large administrative healthcare databases. Prior research has consistently demonstrated that starting in adolescence, depressive and anxiety disorders have higher prevalence rates among females (24). We therefore also evaluated for sex-specific differences in the prevalence of depressive disorders, anxiety disorders and antidepressant prescriptions among adolescent and young adults with CAH.

## Materials and methods

### Data

We utilized the Merative™ MarketScan® Commercial and Multi-State Medicaid Research Databases to identify eligible patients. Administrative databases contain records generated as byproducts of reporting or paying for services and do not contain patient-reported or clinical information. We reviewed health insurance encounter records from October 1, 2015 through December 31, 2019, including data on outpatient and inpatient services and filled outpatient pharmacy prescriptions. Data from both databases were accessed and tabulated at the Centers for Disease Control and Prevention (CDC) using Merative MarketScan Treatment Pathways, an online analytic platform that is licensed to CDC and restricted to health plans that report outpatient pharmacy records for their enrollees, i.e., no pharmacy carve-outs. Data from the Commercial database relate to employees and their dependents enrolled in participating employer-sponsored health insurance plans throughout the United States. The Medicaid database includes data for children, adolescents and young adults enrolled in Medicaid or Children’s Health Insurance programs from participating states, varying in number from 6 to 13 states.

We restricted both the Commercial and Medicaid samples to plans that report mental health encounters for their enrollees, i.e., no mental health carve-outs. We included records from all health plans, both capitated plans, which report records of services provided during encounters despite not filing claims for reimbursement, and non-capitated or fee-for-service plans with billing claims; by convention, we refer to both types of encounter records as claims.

Patients in eligible plans were included if they were enrolled at any point during October 1, 2015 through December 31, 2019, with no minimum length of enrollment specified, and had one or more claims during that period. For eligible patients, recorded age in years and sex were abstracted from the database at the start of the study period. We initially evaluated four age groups: preschool-aged children (3-5 years), school-aged children (6-11 years), adolescents (12-17 years), and young adults (18-25 years). Due to the small number of eligible patients aged 3 to 5 years, we created a single group of eligible children 4-11 years old, after excluding those with an age of 3 years.

Individuals who met the case definitions for mental health diagnoses or prescriptions were included in the age-specific prevalence calculations if they remained within the same age group at the time of the first mental health diagnostic or prescription claim. For example, a child who was initially aged 10 and became diagnosed with depression at age 12 is not included in the calculation of the prevalence of depression for the 4-11 age group. The denominators for those calculations were the numbers of people in the age group at the start of the period. Sex was defined as “Male” or “Female” but is not specified in either database as referring specifically to biological sex versus self-identified gender. No demographic variables other than age and sex were available in both databases.

### Congenital adrenal hyperplasia

We defined Individuals with CAH using a previously-defined algorithm for pediatric CAH cases based on diagnostic codes from the International Statistical Classification of Diseases and Related Health Problems, Tenth Revision, Clinical Modification (ICD-10-CM) as well as prescription drug data (25). Individuals were classified as having CAH if they had at least one claim with the ICD-10-CM code E25.0 (“Congenital adrenogenital disorders associated with enzyme deficiency”) in any setting, no ICD-10 claims with a diagnosis code for a pituitary disorder (ICD-10: E228.x, E229.x, E236.x, E237.x), and at least two filled prescriptions for a glucocorticoid, with the second fill at least 28 days after and within 365 days of the first fill. The prevalence of CAH was defined as the percentage of children, adolescents, and young adults meeting these criteria.

### Depressive and anxiety disorders

We classified individuals as having a “depressive disorder” if they had two or more outpatient claims or encounters at least 1 week apart or at least one claim from an inpatient setting with an ICD-10 diagnosis code for a depressive disorder (F32.0-32.4, F32.8, F32.9, F33.0-33.41, F33.8, F33.9, F34.1, F34.9, F43.21, F43.23, O90.6). We classified individuals as having an “anxiety disorder” if they had two or more outpatient claims a week apart or at least one claim from an inpatient setting with an ICD-10 diagnosis code for an anxiety disorder (F40.0, F40.1, F40.2, F40.8, F40.9, F41.0-41.3, F41.8, F41.9, F43.22, F43.23, F93.0). Specific ICD-10 codes were identified through review of the published literature (17, 26–42) and a methodology for syndrome surveillance established by the Centers for Disease Control and Prevention (43). This comprehensive list of ICD-10 codes was refined through expert review for application to the pediatric population.

### Antidepressant prescriptions

Individuals met the criteria for a filled antidepressant prescription if they had at least one diagnosis code for either a depressive or anxiety disorder, as defined above, and two or more outpatient pharmacy filled prescriptions for an antidepressant medication separated by at least 14 days with no maximum (see **Supplemental Table 1** for the list of included medications).

### Statistical analysis

To compare the prevalence of depressive disorders, anxiety disorders, and filled antidepressant prescriptions between the non-CAH and CAH groups, we calculated prevalence differences and prevalence ratios (PRs). We calculated 95% confidence intervals (CIs) for PRs using a Taylor series linearization of estimated variance and p-values for PRs using a two-sided Mantel-Haenszel chi-square test in MATLAB (Mathworks, Natick, MA). Because a few comparisons had expected prevalence numbers below the cutoff of expected numbers for an asymptotic chi-square test we also calculated p-values using an exact test. Almost all p-values were similar in terms of statistical significance above or below a p value of 0.05; exceptions are noted below (results available on request).

Analyses were calculated separately by payer type (Commercial sample and Medicaid sample) with stratification by age group. Owing to the appearance of sex-based differences beyond age 11, we also reported sex-specific estimates for the adolescent (12-17 years) and young adult (18-25 years) groups. For the main effect of CAH in each subsample, Mantel-Haenszel adjusted prevalence ratios (aPRs) are presented after stratification by age. The PRs presented for each age strata are unadjusted. Prevalence data for groups with fewer than five individuals meeting the case definition are not presented.

## Results

Using our claims-based algorithm, we identified a total of 1056 individuals with CAH in the Commercial sample (N=12,313,882) and 570 individuals in the Medicaid sample (N=9,316,824). Descriptive data are presented for each sample in **Tables 1**, **2**, respectively. In the general pediatric and young adult population, mental disorders and antidepressant prescriptions were higher within the Medicaid sample compared with the Commercial sample (**Supplemental Figure 1**). In the general pediatric population not treated for CAH, there was little difference between males and females prior to adolescence, i.e., aged 4-11, in the prevalence of depressive disorders and anxiety disorders (**Supplemental Figure 2**).

**Table 1:** Commercial insurance sample demographics based on congenital adrenal hyperplasia (CAH) diagnosis and the presence of a mental disorder diagnosis or a filled antidepressant prescription.

	All		Depressive Disorder^1^			Anxiety Disorder^2^			Antidepressant Prescription^3^		
	Non-CAH n	CAH n	Non-CAH Cases (%)	CAH Cases (%)	PD	Non-CAH Cases (%)	CAH Cases (%)	PD	Non-CAH Cases (%)	CAH Cases (%)	PD
4-11 y	3,935,932	370	60,867 (1.5)	11 (3.0)	1.4	167,453 (4.3)	28 (7.6)	3.3	49,250 (1.3)	7 (1.9)	0.6
12-17 y	3,262,212	313	254,529 (7.8)	34 (10.9)	3.1	308,607 (9.5)	45 (14.4)	4.9	227,867 (7.0)	29 (9.3)	2.3
18-25 y	5,114,682	373	396,310 (7.7)	54 (14.5)	6.7	532,236 (10.4)	66 (17.7)	7.3	487,041 (9.5)	68 (18.2)	8.7
*Males*											
12-17 y	1,628,564	129	91,603 (5.6)	16 (12.4)	6.8,	168,895 (10.4)	17 (13.2)	2.8	80,125 (4.9)	13 (10.1)	5.2
18-25 y	2,266,275	123	141,147 (6.2)	15 (12.2)	6.0	281,249 (12.4)	16 (13.0)	0.6	156,173 (6.9)	19 (15.4)	8.6
*Females*											
12-17 y	1,633,648	184	162,926 (10.0)	18 (9.8)	-0.2	139,712 (8.6)	28 (15.5)	6.7	147,742 (9.0)	16 (8.7)	-0.3
18-25 y	2,848,407	250	255,163 (9.0)	39 (15.6)	6.6	250,987 (8.9)	50 (20.0)	11.2	330,868 (11.6)	49 (19.6)	8.0

Years (y). prevalence difference (PD), expressed as percentage points. ^1^Defined as 2 or more outpatient claims or 1 or more inpatient claims for a depressive disorder. ^2^Defined as 2 or more outpatient claims or 1 or more inpatient claims for an anxiety disorder. ^3^Defined as at least 2 filled antidepressant prescriptions and at least 1 claim for either a depressive or anxiety disorder.

**Table 2:** Medicaid sample demographics based on congenital adrenal hyperplasia (CAH) diagnosis and the presence of a mental disorder diagnosis or a filled antidepressant prescription.

	All		Depressive Disorder^1^			Anxiety Disorder^2^			Antidepressant Prescription^3^		
	Non-CAH n	CAH n	Non-CAH Cases (%)	CAH Cases (%)	PD	Non-CAH Cases (%)	CAH Cases (%)	PD	Non-CAH Cases (%)	CAH Cases (%)	PD
4-11 y	2,956,016	208	104,316 (3.5)	26 (12.5)	9.0	166,825 (5.6)	23 (11.1)	5.4	64,708 (2.2)	13 (6.3)	4.1
12-17 y	1,836,169	115	224,997 (12.3)	27 (23.5)	11.2	189,213 (10.3)	24 (20.9)	10.6	168,435 (9.2)	25 (21.7)	12.6
18-25 y	1,343,950	66	156,853 (11.7)	15 (22.7)	11.1	160,306 (11.9)	15 (22.7)	10.8	145,926 (10.9)	17 (25.8)	14.9
*Males*											
12-17 y	914,446	44	79,866 (8.7)	9 (20.5)	11.7	66,702 (7.3)	8 (18.2)	10.9	60,335 (6.6)	9 (20.5)	12.0
18-25 y	364,496	21	36,909 (10.1)	–	–	36,707 (10.1)	–	–	33,023 (9.1)	–	–
*Females*											
12-17 y	921,723	71	145,131 (15.7)	18 (25.4)	9.6	122,511 (13.3)	16 (22.5)	9.2	108,100 (11.7)	16 (22.5)	10.8
18-25 y	979,454	45	119,944 (12.2)	15 (33.3)	21.1	123,599 (12.6)	14 (31.5)	18.5	112,903 (11.5)	15 (33.3)	21.8

Years (y). prevalence difference (PD), expressed as percentage points. Data not presented, <5 cases in subgroup (–). ^1^Defined as 2 or more outpatient claims or 1 inpatient claim for a depressive disorder. ^2^Defined 2 or more outpatient claims or 1 inpatient claim for an anxiety disorder. ^3^Defined as at least 2 filled antidepressant prescriptions and at least 1 claim for either a depressive or anxiety disorder. Males with CAH in the Medicaid sample aged 18-25 years were excluded from this analysis as fewer than five subjects met the case definitions for depressive disorders, anxiety disorders, or antidepressant prescriptions.

**Figure 1:**
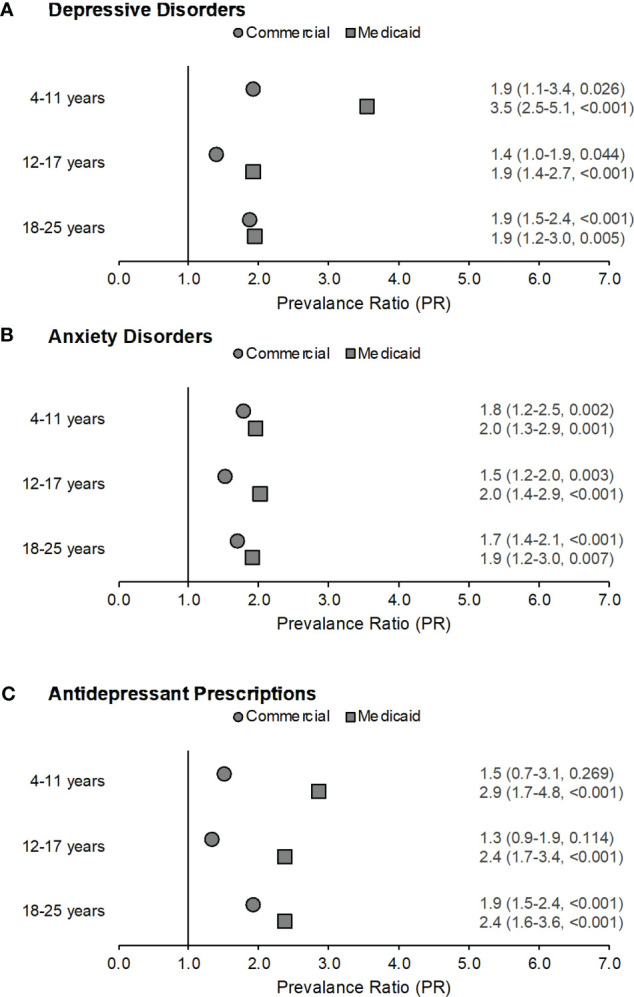
Prevalence ratios by age across both samples for depressive disorders (part **A**), anxiety disorders (part **B**), and filled antidepressant prescriptions (part **C**) among those with congenital adrenal hyperplasia (CAH) as compared to non-CAH peers in the same age group. Data labels reflect prevalence ratio (95% confidence interval, p-value). Males with CAH in the Medicaid sample ages 18-25 years were excluded from this analysis as fewer than five subjects met the case definitions for depressive disorders, anxiety disorders, and antidepressant prescriptions.

Within the Commercial sample, depressive disorders (**Figure 1A**) were significantly more likely among children (PR=1.9, 95% CI:1.1-3.4, p=0.026), adolescents (PR=1.4, 95% CI:1.0-1.9, p=0.044), and young adults (PR=1.9, 95% CI:1.5-2.4, p<0.001) with CAH when compared with their non-CAH peers in the same age group using the Mantel-Haenszel chi square test. However, the differences for the 4-11 and 12-17 years age groups were not significant when calculated using a Fisher exact test. Anxiety disorders (**Figure 1B**) were also more likely among children (PR=1.8, 95% CI:1.2-2.5, p=0.002), adolescents (PR=1.5, 95% CI:1.2-2.0, p=0.003), and young adults (PR=1.7, 95% CI:1.4-2.1, p<0.001) with CAH when compared with their non-CAH peers. Filled antidepressant prescriptions were significantly elevated only among young adults with CAH (PR=1.9, 95% CI:1.5-2.4, p<0.001) (**Figure 1C**).

Within the Medicaid sample, depressive disorders (**Figure 1A**) were more likely among children (PR=3.5, 95% CI:2.5-5.1, p<0.001), adolescents (PR=1.9, 95% CI:1.4-2.7, p<0.001), and young adults (PR=1.9, 95% CI:1.2-3.0, p=0.005) with CAH as compared with their non-CAH peers. Anxiety disorders (**Figure 1B**) were also more likely among children (PR=2.0, 95% CI:1.3-2.9, p=0.001), adolescents (PR=2.0, 95% CI:1.4-2.9, p<0.001), and young adults (PR=1.9, 95% CI:1.2-3.0, p=0.007) with CAH when compared with their non-CAH peers. The prevalence of filled antidepressant prescriptions (**Figure 1C**) was also elevated among individuals with CAH for all age groups (children: PR=2.9, 95% CI:1.7-4.8, p<0.001; adolescents: PR=2.4, 95% CI:1.7-3.4, p<0.001; young adults: PR=2.4, 95% CI:1.6-3.6, p<0.001) as compared to their peers without CAH.

**Figure 2:**
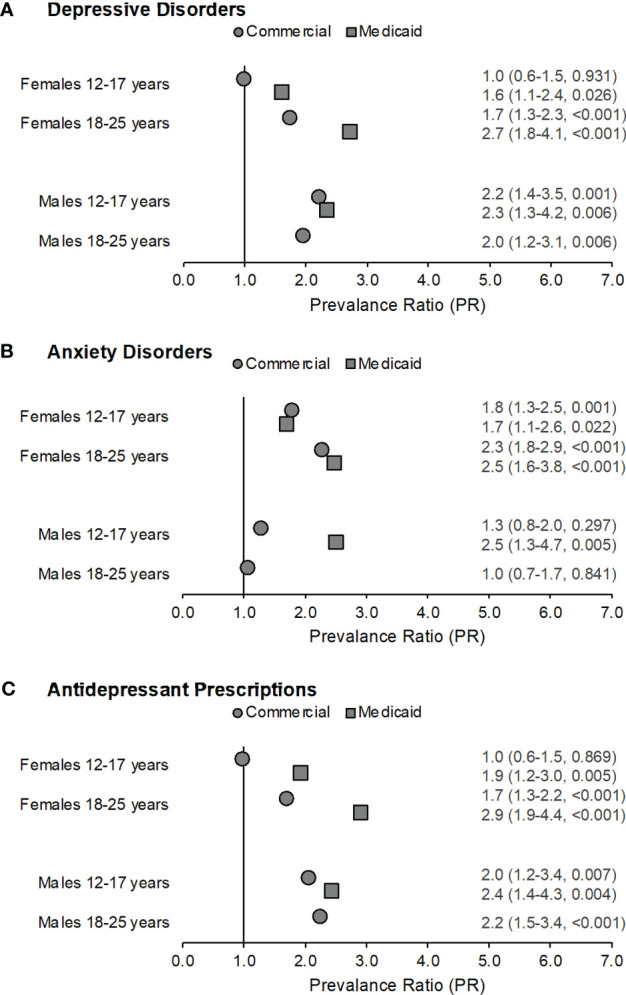
Prevalence ratios by gender across both samples for depressive disorders (part **A**), anxiety disorders (part **B**), and filled antidepressant prescriptions (part **C**) among those with congenital adrenal hyperplasia (CAH) as compared to their non-CAH peers. Data labels reflect prevalence ratio (95% confidence interval, p-value). Males with CAH in the Medicaid sample aged 18-25 years were excluded from this analysis as fewer than five subjects met the case definitions for depressive disorders, anxiety disorders, or antidepressant prescriptions.

We next compared prevalence ratios for depressive disorders, anxiety disorders, and filled antidepressant prescriptions among adolescents and young adults after stratifying by sex. Within the Commercial sample, adolescent females with CAH were more likely to have an anxiety disorder than adolescent females without CAH (PR=1.8, 95% CI:1.3-2.5, p=0.001), but not a depressive disorder or filled antidepressant prescription (**Figure 2**). Young adult females with CAH were more likely to have a depressive disorder (PR=1.7, 95% CI:1.3-2.3, p<0.001) or an anxiety disorder (PR=2.3, 95% CI:1.8-2.9, p<0.001), and to fill a prescription for an antidepressant (PR=1.7, 95% CI:1.3-2.2, p<0.001) compared to young adult females without CAH.

Adolescent and young adult males in the Commercial sample were approximately twice as likely to have a depressive disorder (adolescents: PR=2.2, 95% CI:1.4-3.5, p=0.001; young adults: PR=2.0, 95% CI:1.2-3.1, p=0.006) and to fill a prescription for an antidepressant (adolescents: PR=2.0, 95% CI:1.2-3.4, p=0.007; young adults: PR=2.2, 95% CI:1.5-3.4, p<0.001) compared to adolescent and young adult males without CAH. There were no significant differences for anxiety disorders among adolescent or young adult males.

Adolescent females with CAH in the Medicaid sample were 1.6-1.9 times as likely to have a depressive disorder (PR=1.6, 95% CI:1.1-2.4, p=0.026) or an anxiety disorder (PR=1.7, 95% CI:1.1-2.6, p=0.022), and fill a prescription for an antidepressant (PR=1.9, 95% CI:1.2-3.0, p=0.005) as compared to adolescent females without CAH (**Figure 2**). Young adult females with CAH were 2.5-2.9 times as likely to have a depressive disorder (PR=2.7, 95% CI:1.8-4.1, p<0.001) or an anxiety disorder (PR=2.5, 95% CI:1.6-3.8, p<0.001), and to fill a prescription for an antidepressant (PR=2.9, 95% CI:1.9-4.4, p<0.001) as compared to young adult females without CAH.

The prevalence ratio was 2.3-2.5 times higher for adolescent males with CAH in the Medicaid sample for a depressive disorder (PR=2.3, 95% CI:1.3-4.2, p=0.006) or an anxiety disorder (PR=2.5, 95% CI: 1.3-4.7, p=0.005), and a filled antidepressant prescription (PR=2.4, 95% CI:1.4-4.3, p=0.004) as compared to adolescent males without CAH. Because fewer than five young adult males in the Medicaid sample with CAH met the case definitions for depressive disorders, anxiety disorders, or antidepressant prescriptions, results for this group were not reported (**Table 2**).

## Discussion

In this retrospective cohort study, we examined the administrative prevalence of diagnosed depressive disorders, anxiety disorders, and filled antidepressant prescriptions among children, adolescents, and young adults with and without CAH using two large administrative healthcare databases. Depression and anxiety are common mental disorders that often emerge in late childhood or adolescence and can result in significant disability (44, 45). If untreated, symptoms that begin during childhood and adolescence may recur later in life (46, 47) and can lead to long-term functional impairment in adulthood (48). Thus, determining whether the risks for these conditions are elevated among the pediatric and young adult CAH population is important to improve clinical care for these individuals.

School-aged children, adolescents, and young adults with CAH were relatively more likely to have records of a depressive disorder or an anxiety disorder compared to their peers without a CAH diagnosis in both the commercially and publicly insured samples (**Figure 1**). The finding of a lower administrative prevalence of the studied disorders prior to age 11 with no variation by sex (**Supplementary Figure 2**) is consistent with published evidence that male-female differences in depressive and anxiety disorders emerge in early adolescence (with consistently higher prevalence rates of depression and anxiety in female adolescents) (24). Also consistent with prior research in the general pediatric and young adult population (49), we found that the absolute administrative prevalence estimates for depressive disorders and anxiety disorders among individuals with and without CAH increased from childhood to adolescence (**Tables 1**, **2**).

Our findings extend those of other large studies of CAH in children, adolescents, and young adults. In particular, In particular, Sewell and colleagues (50) reported no increased risk for depressive disorders among a cohort of 1,647 patients under the age of 18 years with a diagnosis of CAH recorded in electronic health records at one of six U.S. children’s hospitals who had at least one outpatient visit during 2009–2019. In the same pediatric population, the investigators also found no increased risk for anxiety among males under the age of 18 with CAH, and lower odds of anxiety for females under the age of 18 with CAH compared with females without CAH (odds ratio = 0.7). The differences between our findings and Sewell’s finding may reflect differences in classification of CAH case status. Unlike our study, Sewell et al. did not restrict their analysis to individuals with treated CAH. That might have biased associations in their study towards the null if untreated individuals with diagnostic codes for “congenital adrenogenital disorders associated with enzyme deficiencies” did not actually have CAH. In our sample, fewer than half of individuals with a diagnosis code for CAH had a minimum of two filled prescriptions for glucocorticoids.

In our study, sex differences in the relative prevalence of diagnosed depressive and anxiety disorders and filled antidepressant prescriptions among adolescents with CAH were attenuated compared to the general population. Adolescent males with CAH had much higher rates of the mental health outcomes than their non-CAH male agemates. Prevalence ratios for depressive disorder and antidepressants were slightly higher for young adult males than for females in the Commercial sample; results were not reported for the Medicaid sample as noted in the Methods section. Anxiety disorders were more common for females in both age groups in both samples as well as for Medicaid-enrolled male adolescents (**Figure 2**). Commercially insured young adults with CAH as well as publicly insured children, adolescents, and young women with CAH were also approximately twice as likely to fill prescriptions for an antidepressant (**Figures 1**, **2**). The findings of increased prevalence of filled antidepressant prescriptions among CAH patients relative to the general population are consistent with data from the UK Clinical Practice Research Datalink (17). This speaks to the impact and burden of depressive disorders in this population.

The higher absolute administrative prevalence estimates for depressive and anxiety disorders observed in the Medicaid sample may be in part explained by the Medicaid qualification process. Mood and anxiety disorders can be qualifying conditions for disability benefits under the Supplemental Security Income program, which in turn makes individuals eligible for Medicaid (51). Many children also qualify for Medicaid coverage based on household income, and the prevalence of mental, behavioral and development disorders among children living in lower-income households in the United States is higher than among those in higher-income households (52). Children and young adults with public insurance may also be at greater risk for adverse childhood experiences, such as witnessing violence and trauma, relative to those with private insurance. That would in turn raise their risk for negative health outcomes, including mental disorders (i.e., the toxic stress hypothesis) (53). The higher prevalence ratios for depressive disorders, anxiety disorders, and filled antidepressant prescriptions seen in some CAH groups in the Medicaid sample as compared to the Commercial sample could suggest that the presence of CAH may interact with or enhance these risks.

The pattern of sex-based differences in the prevalence of depressive disorders, anxiety disorders, and filled antidepressant prescriptions among adolescents and young adults with CAH was somewhat unexpected. While depression and anxiety are more commonly diagnosed among females than males beginning in adolescence (24, 54–56), the findings indicate that the likelihood of meeting criteria for a depressive or anxiety disorder or filling an antidepressant prescription among males with CAH was comparable to or higher than among females with CAH in the same age groups, except for anxiety among publicly insured males with CAH. These findings suggest a narrowing of the gender gap in depressive disorders and anxiety disorders among youth with CAH. This is consistent with data from the UK Clinical Practice Research Datalink (17), which showed a substantially narrower gender gap in depression diagnoses or antidepressant prescriptions in children or adolescents with CAH than among the general population, with a higher prevalence ratio relative to population controls for males with CAH (PR=2.2) than females (PR=1.2).

Pediatric and young adult patients with CAH may be at greater risk for mood and anxiety disorders due to multiple mechanisms. First, the burdens of living with a chronic disease, such as CAH, including recommended daily medication adherence and frequent healthcare contacts, may increase children’s risk for anxiety and depression. Multiple studies have shown an increased risk for anxiety and depression among children with a wide range of chronic physical illnesses (57, 58) and life-limiting conditions (59). Second, children and young adults with CAH may be at increased risk for depression and anxiety due to the specific disease pathology and side effects from treatment. Dysfunction in the hypothalamic-pituitary-adrenal (HPA) axis and hypothalamic-pituitary-gonadal axis is known to impact mental health (60–62), and in particular, may alter stress reactivity and lead to downstream effects on mood and anxiety. In CAH patients, hypocortisolemia disrupts the endogenous negative feedback loop of the HPA axis and leads to overproduction of adrenal androgens. Further, cortisol replacement with glucocorticoids is unable to fully replicate the circadian and ultradian cortisol secretion rhythms associated with normal adrenal function (63). In a recent clinical trial utilizing a block and replace design among healthy young adult volunteers to test the significance of glucocorticoid replacement rhythmicity in mood regulation and neural dynamics, oral glucocorticoid replacement three times a day (which is the standard of care for CAH patients) showed a decrease in positive mood and an increase in negative mood throughout the day (64). This contrasted with individuals undergoing pulsatile glucocorticoid replacement (which more closely approximated physiological ultradian cortisol rhythms), in which mood variation more closely approximates what is thought to be normal variation in daily mood. Thus, excessive adrenal androgens, fluctuating cortisol levels, or over-suppression of the HPA axis could contribute to the development of depressive and anxiety disorders among CAH patients.

To elucidate potential causal pathways from CAH to prevalence of depression and anxiety, future work might examine the extent to which these associations vary relative to patients’ specific enzyme defects, disease severity and phenotype, glucocorticoid form and dosage, and medication adherence. For example, the two Swedish registry-based studies assessed disease severity and phenotype. One study reported that the odds ratio for mood disorders was 2.0 among males with salt-wasting phenotypes vs 1.1 among those with the simple virilizing form, while the other study found no difference among female patients (18, 19). Jenkins-Jones and colleagues reported lower medication adherence among adult patients than pediatric patients but did not assess how adherence was related to anxiety and depression (17). The present study is limited in that it did not assess medication possession ratios.

There are several additional limitations to our study. Despite the overall large number of individuals with CAH identified in our study, we could not include young adult males in the Medicaid sample Diagnosis codes for medical and mental health conditions in claims and encounters data are subject to miscoding or incomplete coding, which may have resulted in individual misclassification. We sought to minimize the impact of miscoding, which is more common in outpatient records, by using algorithms that require diagnosis codes be present on at least two outpatient records on different dates. Further, while the current results build upon our prior study detailing the development of our CAH case algorithm, the sensitivity and specificity of our CAH algorithm has yet to be validated through comparison with external data sources, such as medical records. Additionally, because Treatment Pathways reports age in years, we used broad age groups. Because we excluded mental health diagnoses for individuals who moved between age groups during the 3-year study period, we did not include all cases of mental health diagnoses in our age-specific prevalence estimates. Cumulative age-specific prevalence estimates without exclusion of those who aged out were higher for both the CAH and non-CAH populations, especially for the youngest age group, but prevalence ratios were almost all within 10% of those reported (results not reported).

The MarketScan datasets are convenience samples and hence the findings may not be generalizable to the populations of people with employer-sponsored or Medicaid insurance. Finally, we were unable to control for risk factors and cofounders not included in both databases, such as race and ethnicity (included in Medicaid only), geographical location (included in Commercial only), stated gender identity versus biological sex, parent education level, and socioeconomic status (neither database). Additional research using other data sources could potentially elucidate the impact of these factors.

In conclusion, in our retrospective cohort study we found that children, adolescents, and young adults with CAH in the United States were more likely to be diagnosed with a depressive or anxiety disorder and to be prescribed antidepressants as compared to their age and sex matched peers. The likelihood of these conditions increased with age and did not follow the same gender distribution commonly observed in the pediatric and young adult population, with a concentration of cases among males with CAH. If these associations are confirmed in further research, enhanced screening for symptoms of depression and anxiety among the pediatric and young adult population with CAH may be warranted.

## Data availability statement

The datasets analyzed for this study are the property of Merative (Ann Arbor, Michigan, USA), formerly IBM Watson Health, and may be accessed by researchers through a contract and data use agreement with the company.

## Author contributions

LH, SD, and KS contributed to the study conception and design. Data analysis was performed by LH and SD. The first draft of the manuscript was written by LH.SD and KS wrote sections of the manuscript. KC, RB and RB contributed to manuscript revision and additional data interpretation. All authors read, and approved the submitted version. All authors contributed to the article and approved the submitted version.

## References

[1] Merke DP Auchus RJ. Congenital adrenal hyperplasia due to 21-hydroxylase deficiency. N Engl J Med (2020) 383(13):1248–61. doi: 10.1056/NEJMra1909786 32966723

[2] Sarafoglou K Reiner W Hindmarsh PC. Congenital adrenal hyperplasia. In: Sarafoglou K Hoffmann GF Roth KS, editors. Pediatric Endocrinology and Inborn Errors of Metabolism, 2e. New York, NY: McGraw-Hill Education (2017). p. 533–59.

[3] Speiser PW Arlt W Auchus RJ Baskin LS Conway GS Merke DP. Congenital adrenal hyperplasia due to steroid 21-hydroxylase deficiency: An Endocrine Society clinical practice guideline. J Clin Endocrinol Metab (2018) 103(11):4043–88. doi: 10.1210/jc.2018-01865 PMC6456929 30272171

[4] Therrell BL Jr. Berenbaum SA Manter-Kapanke V Simmank J Korman K Prentice L. Results of screening 1.9 million Texas newborns for 21-hydroxylase-deficient congenital adrenal hyperplasia. Pediatrics (1998) 101(4 Pt 1):583–90. doi: 10.1542/peds.101.4.583 9521938

[5] Pearce M DeMartino L McMahon R Hamel R Maloney B Stansfield DM. Newborn screening for congenital adrenal hyperplasia in New York State. Mol Genet Metab Rep (2016) 7:1–7. doi: 10.1016/j.ymgmr.2016.02.005 27331001 PMC4908061

[6] Hannah-Shmouni F Morissette R Sinaii N Elman M Prezant TR Chen W. Revisiting the prevalence of nonclassic congenital adrenal hyperplasia in US Ashkenazi Jews and Caucasians. Genet Med (2017) 19(11):1276–9. doi: 10.1038/gim.2017.46 PMC5675788 28541281

[7] Al-Kofahi M Ahmed MA Jaber MM Tran TN Willis BA Zimmerman CL. An integrated PK-PD model for cortisol and the 17-hydroxyprogesterone and androstenedione biomarkers in children with congenital adrenal hyperplasia. Br J Clin Pharmacol (2021) 87(3):1098–110. doi: 10.1111/bcp.14470 PMC9328191 32652643

[8] Sarafoglou K Gonzalez-Bolanos MT Zimmerman CL Zimmerman CL Boonstra T Yaw Addo O. Comparison of cortisol exposures and pharmacodynamic adrenal steroid responses to hydrocortisone suspension vs. commercial tablets. J Clin Pharmacol (2015) 55(4):452–7. doi: 10.1002/jcph.424 25385533

[9] Han TS Walker BR Arlt W Ross RJ. Treatment and health outcomes in adults with congenital adrenal hyperplasia. Nat Rev Endocrinol (2014) 10(2):115–24. doi: 10.1038/nrendo.2013.239 24342885

[10] Sarafoglou K Addo OY Turcotte L Otten N Wickremasinghe A Pittock S. Impact of hydrocortisone on adult height in congenital adrenal hyperplasia-the Minnesota cohort. J Pediatr (2014) 164(5):1141–6. doi: 10.1016/j.jpeds.2014.01.011 24560184

[11] Al-Rayess H Fleissner K Jaber M Brundage RC Sarafoglou K. Manipulation of hydrocortisone tablets leads to iatrogenic cushing syndrome in a 6-year-old girl with CAH. J Endocr Soc (2020) 4(8):bvaa091. doi: 10.1210/jendso/bvaa091 32803093 PMC7417883

[12] Maccabee-Ryaboy N Thomas W Kyllo J Lteif A Petryk A Gonzalez-Bolanos MT. Hypertension in children with congenital adrenal hyperplasia. Clin Endocrinol (Oxf). (2016) 85(4):528–34. doi: 10.1111/cen.13086 27105393

[13] Sarafoglou K Forlenza GP Yaw Addo O Kyllo J Lteif A Hindmarsh PC. Obesity in children with congenital adrenal hyperplasia in the Minnesota cohort: importance of adjusting body mass index for height-age. Clin Endocrinol (Oxf). (2017) 86(5):708–16. doi: 10.1111/cen.13313 PMC6006505 28199739

[14] Muthusamy K Elamin MB Smushkin G Murad MH Lampropulos JF Elamin KB. Clinical review: Adult height in patients with congenital adrenal hyperplasia: a systematic review and metaanalysis. J Clin Endocrinol Metab (2010) 95(9):4161–72. doi: 10.1210/jc.2009-2616 20823467

[15] Arlt W Willis DS Wild SH Krone N Doherty EJ Hahner S. Health status of adults with congenital adrenal hyperplasia: A cohort study of 203 patients. J Clin Endocrinol Metab (2010) 95(11):5110–21. doi: 10.1210/jc.2010-0917 PMC3066446 20719839

[16] Gilban DLS Alves Junior PAG Beserra ICR. Health related quality of life of children and adolescents with congenital adrenal hyperplasia in Brazil. Health Qual Life outcomes. (2014) 12:107. doi: 10.1186/s12955-014-0107-2 25115634 PMC4243953

[17] Jenkins-Jones S Parviainen L Porter J Withe M Whitaker MJ Holden SE. Poor compliance and increased mortality, depression and healthcare costs in patients with congenital adrenal hyperplasia. Eur J Endocrinol (2018) 178(4):309–20. doi: 10.1530/EJE-17-0895 29371334

[18] Engberg H Butwicka A Nordenstrom A Hirschberg AL Falhammar H Lichtenstein P. Congenital adrenal hyperplasia and risk for psychiatric disorders in girls and women born between 1915 and 2010: A total population study. Psychoneuroendocrinology (2015) 60:195–205. doi: 10.1016/j.psyneuen.2015.06.017 26184920

[19] Falhammar H Butwicka A Landen M Lichtenstein P Nordenskjold A Nordenstrom A. Increased psychiatric morbidity in men with congenital adrenal hyperplasia due to 21-hydroxylase deficiency. J Clin Endocrinol Metab (2014) 99(3):E554–60. doi: 10.1210/jc.2013-3707 24302749

[20] Messina V Hirvikoski T Karlsson L Vissani S Wallensteen L Ortolano R. Good overall behavioural adjustment in children and adolescents with classic congenital adrenal hyperplasia. Endocrine (2020) 68(2):427–37. doi: 10.1007/s12020-020-02244-1 PMC7266840 32152914

[21] Kung KTF Spencer D Pasterski V Neufeld SAS Hindmarsh PC Hughes IA. Emotional and behavioral adjustment in 4 to 11-year-old boys and girls with classic congenital adrenal hyperplasia and unaffected siblings. Psychoneuroendocrinology (2018) 97:104–10. doi: 10.1016/j.psyneuen.2018.07.004 30015005

[22] Idris AN Chandran V Syed Zakaria SZ Rasat R. Behavioural outcome in children with congenital adrenal hyperplasia: experience of a single centre. Int J Endocrinol (2014) 2014:483718. doi: 10.1155/2014/483718 24799898 PMC3995314

[23] Berenbaum SA Korman Bryk K Duck SC Resnick SM. Psychological adjustment in children and adults with congenital adrenal hyperplasia. J Pediatr (2004) 144(6):741–6. doi: 10.1016/j.jpeds.2004.03.037 15192620

[24] Hankin BL Abramson LY Moffitt TE Silva PA McGee R Angell KE. Development of depression from preadolescence to young adulthood: emerging gender differences in a 10-year longitudinal study. J Abnorm Psychol (1998) 107(1):128–40. doi: 10.1037/0021-843X.107.1.128 9505045

[25] Harasymiw LA Grosse SD Sarafoglou K. Attention-deficit/hyperactivity disorder among US children and adolescents with congenital adrenal hyperplasia. J Endocr Soc (2020) 4(12):bvaa152. doi: 10.1210/jendso/bvaa152 33195955 PMC7648384

[26] Alaghehbandan R Macdonald D Barrett B Collins K Chen Y. Using administrative databases in the surveillance of depressive disorders–case definitions. Popul Health Manage (2012) 15(6):372–80. doi: 10.1089/pop.2011.0084 22788998

[27] Boulanger L Zhao Y Bao Y Russell MW. A retrospective study on the impact of comorbid depression or anxiety on healthcare resource use and costs among diabetic neuropathy patients. BMC Health Serv Res (2009) 9:111. doi: 10.1186/1472-6963-9-111 19566952 PMC2719623

[28] Diaz I Thurm C Hall M Auerbach S Bearl DW Dodd DA. Disorders of adjustment, mood, and anxiety in children and adolescents undergoing heart transplantation and the association of ventricular assist device support. J Pediatr (2020), 217:20–4.e1. doi: 10.1016/j.jpeds.2019.10.022 31732131

[29] Doktorchik C Patten S Eastwood C Peng M Chen G Beck CA. Validation of a case definition for depression in administrative data against primary chart data as a reference standard. BMC Psychiatry (2019) 19(1):9. doi: 10.1186/s12888-018-1990-6 30616546 PMC6323719

[30] Edwards J Thind A Stranges S Chiu M Anderson KK. Concordance between health administrative data and survey-derived diagnoses for mood and anxiety disorders. Acta Psychiatr Scand (2020) 141(4):385–95. doi: 10.1111/acps.13143 31883386

[31] Fiest KM Jette N Quan H St Germaine-Smith C Metcalfe A Patten SB. Systematic review and assessment of validated case definitions for depression in administrative data. BMC Psychiatry (2014) 14:289. doi: 10.1186/s12888-014-0289-5 25322690 PMC4201696

[32] Greenberg PE Fournier AA Sisitsky T Pike CT Kessler RC. The economic burden of adults with major depressive disorder in the United States (2005 and 2010). J Clin Psychiatry (2015) 76(2):155–62. doi: 10.4088/JCP.14m09298 25742202

[33] Howren A Avina-Zubieta JA Puyat JH Esdaile JM Da Costa D De Vera MA. Defining depression and anxiety in individuals with rheumatic diseases using administrative health databases: A systematic review. Arthritis Care Res (Hoboken). (2020) 72(2):243–55. doi: 10.1002/acr.24048 31421021

[34] Kimball AB Wu EQ Guerin A Yu AP Tsaneva M Gupta SR. Risks of developing psychiatric disorders in pediatric patients with psoriasis. J Am Acad Dermatol (2012) 67(4):651–7.e1-2. doi: 10.1016/j.jaad.2011.11.948 22243764

[35] Kisely S Lin E Gilbert C Smith M Campbell LA Vasiliadis HM. Use of administrative data for the surveillance of mood and anxiety disorders. Aust N Z J Psychiatry (2009) 43(12):1118–25. doi: 10.3109/00048670903279838 20001410

[36] Marrie RA Fisk JD Yu BN Leung S Elliott L Caetano P. Mental comorbidity and multiple sclerosis: validating administrative data to support population-based surveillance. BMC Neurol (2013) 13:16. doi: 10.1186/1471-2377-13-16 23388102 PMC3599013

[37] Marrie RA Walker JR Graff LA Lix LM Bolton JM Nugent Z. Performance of administrative case definitions for depression and anxiety in inflammatory bowel disease. J Psychosom Res (2016) 89:107–13. doi: 10.1016/j.jpsychores.2016.08.014 27663119

[38] Noyes K Liu H Lyness JM Friedman B. Medicare beneficiaries with depression: comparing diagnoses in claims data with the results of screening. Psychiatr Serv. (2011) 62(10):1159–66. doi: 10.1176/ps.62.10.pss6210_1159 21969642

[39] O'Donnell S Vanderloo S McRae L Onysko J Patten SB Pelletier L. Comparison of the estimated prevalence of mood and/or anxiety disorders in Canada between self-report and administrative data. Epidemiol Psychiatr Sci (2016) 25(4):360–9. doi: 10.1017/S2045796015000463 PMC7137597 26081585

[40] Schroder C Dorks M Kollhorst B Blenk T Dittmann RW Garbe E. Outpatient antidepressant drug use in children and adolescents in Germany between 2004 and 2011. Pharmacoepidemiol Drug Saf. (2017) 26(2):170–9. doi: 10.1002/pds.4138 27868277

[41] Steffen A Thom J Jacobi F Holstiege J Batzing J. Trends in prevalence of depression in Germany between 2009 and 2017 based on nationwide ambulatory claims data. J Affect Disord (2020) 271:239–47. doi: 10.1016/j.jad.2020.03.082 32479322

[42] Tsai MT Erickson SR Cohen LJ Wu CH. The association between comorbid anxiety disorders and the risk of stroke among patients with diabetes: An 11-year population-based retrospective cohort study. J Affect Disord (2016) 202:178–86. doi: 10.1016/j.jad.2016.03.060 27262640

[43] Anderson KN Radhakrishnan L Lane RI Sheppard M DeVies J Azondekon R. Changes and inequities in adult mental health-related emergency department visits during the COVID-19 pandemic in the US. JAMA Psychiatry (2022) 79(5):475–85. doi: 10.1001/jamapsychiatry.2022.0164 PMC8928092 35293958

[44] Craske MG Stein MB. Anxiety. Lancet (2016) 388(10063):3048–59. doi: 10.1016/S0140-6736(16)30381-6 27349358

[45] Malhi GS Mann JJ. Depression. Lancet (2018) 392(10161):2299–312. doi: 10.1016/S0140-6736(18)31948-2 30396512

[46] Ginsburg GS Becker-Haimes EM Keeton C Kendall PC Iyengar S Sakolsky D. Results From the child/adolescent anxiety multimodal extended long-term study (CAMELS): Primary anxiety outcomes. J Am Acad Child Adolesc Psychiatry (2018) 57(7):471–80. doi: 10.1016/j.jaac.2018.03.017 29960692

[47] Johnson D Dupuis G Piche J Clayborne Z Colman I. Adult mental health outcomes of adolescent depression: A systematic review. Depress Anxiety. (2018) 35(8):700–16. doi: 10.1002/da.22777 29878410

[48] Copeland WE Wolke D Shanahan L Costello EJ. Adult functional outcomes of common childhood psychiatric problems: A prospective, longitudinal study. JAMA Psychiatry (2015) 72(9):892–9. doi: 10.1001/jamapsychiatry.2015.0730 PMC4706225 26176785

[49] Ghandour RM Sherman LJ Vladutiu CJ Ali MM Lynch SE Bitsko RH. Prevalence and treatment of depression, anxiety, and conduct problems in US children. J Pediatr (2019) 206:256–67.e3. doi: 10.1016/j.jpeds.2018.09.021 30322701 PMC6673640

[50] Sewell R Buchanan CL Davis S Christakis DA Dempsey A Furniss A. Behavioral health diagnoses in youth with differences of sex development or congenital adrenal hyperplasia compared with controls: A PEDSnet study. J Pediatr (2021) 239:175–81.e2. doi: 10.1016/j.jpeds.2021.08.066 34461062 PMC8604751

[51] Perrin JM Houtrow A Kelleher K Hoagwood K Stein REK Zima B. Supplemental security income benefits for mental disorders. Pediatrics (2016) 138(1):e20160354. doi: 10.1542/peds.2016-0354 27279648

[52] Cree RA Bitsko RH Robinson LR Holbrook JR Danielson ML Smith C. Health care, family, and community factors associated with mental, behavioral, and developmental disorders and poverty among children aged 2-8 years - United States, 2016. MMWR Morb Mortal Wkly Rep (2018) 67(50):1377–83. doi: 10.15585/mmwr.mm6750a1 PMC6342550 30571671

[53] Branco MSS Linhares MBM. The toxic stress and its impact on development in the Shonkoff’s Ecobiodevelopmental Theorical approach. Estudos Psicologia (Campinas). (2018) 35:89–98. doi: 10.1590/1982-02752018000100009

[54] Platt JM Bates L Jager J McLaughlin KA Keyes KM. Is the US gender gap in depression changing over time? A meta-regression. Am J Epidemiol. (2021) 190(7):1190–206. doi: 10.1093/aje/kwab002 PMC8484777 33423055

[55] Mojtabai R Olfson M Han B. National trends in the prevalence and treatment of depression in adolescents and young adults. Pediatrics (2016) 138(6):e20161878. doi: 10.1542/peds.2016-1878 27940701 PMC5127071

[56] Parodi KB Holt MK Green JG Porche MV Koenig B Xuan Z. Time trends and disparities in anxiety among adolescents, 2012-2018. Soc Psychiatry Psychiatr Epidemiol. (2022) 57(1):127–37. doi: 10.1007/s00127-021-02122-9 PMC8183580 34100110

[57] Pinquart M Shen Y. Depressive symptoms in children and adolescents with chronic physical illness: an updated meta-analysis. J Pediatr Psychol (2011) 36(4):375–84. doi: 10.1093/jpepsy/jsq104 21088072

[58] Pinquart M Shen Y. Anxiety in children and adolescents with chronic physical illnesses: a meta-analysis. Acta Paediatr (2011) 100(8):1069–76. doi: 10.1111/j.1651-2227.2011.02223.x 21332786

[59] Barker MM Beresford B Bland M Fraser LK. Prevalence and incidence of anxiety and depression among children, adolescents, and young adults with life-limiting conditions: A systematic review and meta-analysis. JAMA Pediatrics. (2019) 173(9):835–44. doi: 10.1001/jamapediatrics.2019.1712 PMC6618774 31282938

[60] Marceau K Ruttle PL Shirtcliff EA Essex MJ Susman EJ. Developmental and contextual considerations for adrenal and gonadal hormone functioning during adolescence: Implications for adolescent mental health. Dev Psychobiol (2015) 57(6):742–68. doi: 10.1002/dev.21214 PMC4194172 24729154

[61] Guerry JD Hastings PD. In search of HPA axis dysregulation in child and adolescent depression. Clin Child Fam Psychol Rev (2011) 14(2):135–60. doi: 10.1007/s10567-011-0084-5 PMC3095794 21290178

[62] Juruena MF Eror F Cleare AJ Young AH. The Role of early life stress in HPA axis and anxiety. Adv Exp Med Biol (2020) 1191:141–53. doi: 10.1007/978-981-32-9705-0_9 32002927

[63] Young EA Abelson J Lightman SL. Cortisol pulsatility and its role in stress regulation and health. Front Neuroendocrinol. (2004) 25(2):69–76. doi: 10.1016/j.yfrne.2004.07.001 15571755

[64] Kalafatakis K Russell GM Ferguson SG Grabski M Harmer CJ Munafò MR. Glucocorticoid ultradian rhythmicity differentially regulates mood and resting state networks in the human brain: A randomised controlled clinical trial. Psychoneuroendocrinology (2021) 124:105096. doi: 10.1016/j.psyneuen.2020.105096 33296841 PMC7895801

