# Supplementary material for: Depressive and anxiety disorders and antidepressant prescriptions among insured children and young adults with congenital adrenal hyperplasia in the United States
Source: Front Endocrinol (Lausanne). 2023 Aug 17;14:1129584. doi: 10.3389/fendo.2023.1129584 (PMC10470620; doi:10.3389/fendo.2023.1129584)
Supplement: Supplementary file 1 [file DataSheet_1.docx]

Supplementary Material

**Supplementary Table 1. Antidepressant medications**

1. Amitriptyline Hydrochloride
2. Amitriptyline Hydrochloride/Chlordiazepoxide
3. Amitriptyline Hydrochloride/Perphenazine
4. Amoxapine
5. Bupropion Hydrochloride
6. Citalopram Hydrobromide
7. Escitalopram Oxalate
8. Desipramine Hydrochloride
9. Doxepin Hydrochloride
10. Duloxetine Hydrochloride
11. Fluoxetine Hydrochloride
12. Fluoxetine Hydrochloride/Olanzapine
13. Fluvoxamine Maleate
14. Imipramine Hydrochloride
15. Imipramine Pamoate
16. Trimipramine Maleate
17. Isocarboxazid
18. Levomilnacipran Hydrochloride
19. Maprotiline Hydrochloride
20. Milnacipran Hydrochloride
21. Mirtazapine
22. Nefazodone Hydrochloride
23. Paroxetine Hydrochloride
24. Paroxetine Mesylate
25. Protriptyline Hydrochloride
26. Selegiline Hydrochloride
27. Sertraline Hydrochloride
28. Succinic Acid
29. Trazodone Hydrochloride
30. Desvenlafaxine Succinate
31. Venlafaxine Hydrochloride
32. Vilazodone Hydrochloride
33. Vortioxetine Hydrobromide

**Supplemental Figure 1.** Prevalence estimates for depressive disorders, anxiety disorders, and filled antidepressant prescriptions stratified by insurance type in the general pediatric and young adult populations.

**Supplemental Figure 2.** Prevalence of depressive disorders and anxiety disorders in the general pediatric and young adult populations in the Commercial sample stratified by gender and age
